# Content validation of a Critical Appraisal Tool for Reviewing Analgesia Studies (CATRAS) involving subjects incapable of self-reporting pain

**DOI:** 10.1097/PR9.0000000000000670

**Published:** 2018-07-25

**Authors:** Leon N. Warne, Stephan A. Schug, Thierry Beths, Juliana T. Brondani, Jennifer E. Carter, B. Duncan X. Lascelles, Anthea L. Raisis, Sheilah A. Robertson, Paulo V.M. Steagall, Polly M. Taylor, Ted Whittem, Sébastien H. Bauquier

**Affiliations:** a School of Veterinary and Life Sciences, Murdoch University, Perth, Western Australia, Australia; b Melbourne Veterinary School, The University of Melbourne, Werribee, Victoria, Australia; c Discipline of Anaesthesiology and Pain Medicine, Medical School, University of Western Australia, Perth, Western Australia, Australia; d Department of Anaesthesia and Pain Medicine, Royal Perth Hospital, Perth, Western Australia, Australia; e Universidade Estadual Paulista, Botucatu, Brazil; f Department of Clinical Sciences, Comparative Pain Research and Education Centre, College of Veterinary Medicine, North Carolina State University, Raleigh, NC, USA; g Comparative Medicine Institute, College of Veterinary Medicine, North Carolina State University, Raleigh, NC, USA; h Center for Pain Research and Innovation, UNC School of Dentistry, Chapel Hill, NC, USA; i Department of Anesthesiology, Center for Translational Pain Research, Duke University, Durham, NC, USA; j Lap of Love Veterinary Hospice and In-Home Euthanasia, Lutz, FL, USA; k Department of Clinical Sciences, Faculty of Veterinary Medicine, Université de Montréal, Saint-Hyacinthe, QC, Canada; l Animal Pharmacology Research Group of Quebec (GREPAQ), Faculty of Veterinary Medicine, Université de Montréal, Saint-Hyacinthe, QC, Canada; ^m^Taylor Monroe, Ely, United Kingdom

**Keywords:** Critical appraisal tool, Analgesia, Pain

## Abstract

**Introduction:**

This article reports the content validation of a Critical Appraisal Tool designed to Review the quality of Analgesia Studies (CATRAS) involving subjects incapable of self-reporting pain and provide guidance as to the strengths and weakness of findings. The CATRAS quality items encompass 3 domains: level of evidence, methodological soundness, and grading of the pain assessment tool.

**Objectives:**

To validate a critical appraisal tool for reviewing analgesia studies involving subjects incapable of self-reporting pain.

**Methods:**

Content validation was achieved using Delphi methodology through panel consensus. A panel of 6 experts reviewed the CATRAS in 3 rounds and quantitatively rated the relevance of the instrument and each of its quality items to their respective domains.

**Results:**

Content validation was achieved for each item of the CATRAS and the tool as a whole. Item-level content validity index and kappa coefficient were at least greater than 0.83 and 0.81, respectively, for all items except for one item in domain 2 that was later removed. Scale-level content validity index was 97% (excellent content validity).

**Conclusions:**

This 67-item critical appraisal tool may enable critical and quantitative assessment of the quality of individual analgesia trials involving subjects incapable of self-reporting pain for use in systematic reviews and meta-analysis studies.

## Abstract 2

Supplemental Digital Content is Available in the Text.

## 1. Introduction

The scientific literature contains many examples of inconsistencies regarding the analgesic efficacy of treatments for pain. It is the authors' opinion that many of these inconsistencies arise due to variations in the quality and rigour with which each study was designed.

Determining the quality of published studies of analgesic interventions is difficult, particularly those involving animal pain models, hampering efforts to draw meaningful clinical conclusions from published findings and to perform systematic reviews of the literature.^34,40^ Frameworks are needed to ensure greater consistency in experimental design to allow for more accurate comparison of findings.^40^ The Initiative on Methods, Measurement, and Pain Assessment in Clinical Trials (IMMPACT) has developed consensus reviews and recommendations for improving the design, execution, and interpretation of clinical analgesia trials in self-reporting humans.^9,33,48^ Despite this, recommendations for animals, as well as for humans incapable of self-reporting pain are still lacking.

Descriptors of quality currently used to critically appraise scientific literature typically include schemes for assessing the *level of evidence* (LOE) and *methodological soundness*.^2,14^ The LOE for a particular study is assigned according to the study design and its inherent likelihood to exclude bias. Grading of the methodological soundness of a study is typically based on how closely it conforms to established standards for study design. The “2011 Levels of Evidence” established by the Oxford Centre for Evidence-Based Medicine (OCEBM) and the Grading of Recommendations Assessment, Development, and Evaluation (GRADE) system are 2 of the most universally recognised ranking systems.^7,17,19,43^ These critical appraisal tools (CATs) include checklists with specific questions and/or scales for scoring components of quality, which are combined to give a summary score.^24^

Current “gold-standard” pain assessment tools (PATs) rely on self-reporting, requiring an individual subject to both process external information and communicate this personal experience. In relation to subjects incapable of self-reporting pain such as noncommunicative or cognitively impaired human patients or pain in animal, this is not possible. In these situations, the assessment of pain involves changes in behavioural or physiological parameters. However, their use can be associated with considerable shortcomings. They may be unreliable, hampered by observational bias, or influenced by disease processes or pharmacological interventions. Current evidence indicates that treatment of pain is inadequate in human patients incapable of self-reporting pain as well as animal, largely because of inadequate methods of pain assessment.^23,27,32,39,44,52^ This brings into question the accuracy and validity of findings from published studies using non–self-reporting PATs. Several assessment techniques have been designed specifically for the *grading of PATs* in noncommunicative human patients; these primarily evaluate the psychometric properties of PATs against: item selection and content validation, reliability, validity, feasibility, and relevance or impact on patient outcomes.^16,38,52^

Critical appraisal and interpretation of findings from analgesia studies remains challenging because of the lack of a single CAT for evaluating all domains (ie, *LOE*, *methodological soundness*, and *grading of the PATs*). The aim of the present work was to construct and validate a CAT that incorporated the aforementioned 3 domains to assess the quality of individual analgesia trials and provide quantification of quality for use in systematic reviews and meta-analysis studies focusing on subjects incapable of self-reporting pain. Importantly, this CAT will assess whether the methodologies used by a study conform to appropriately high scientific standards, independent of the species being studied. This study reports the content validation of this CAT referred to as “CATRAS” for Critical Appraisal Tool for Reviewing Analgesia Studies.

## 2. Methods

**Figure 1.**
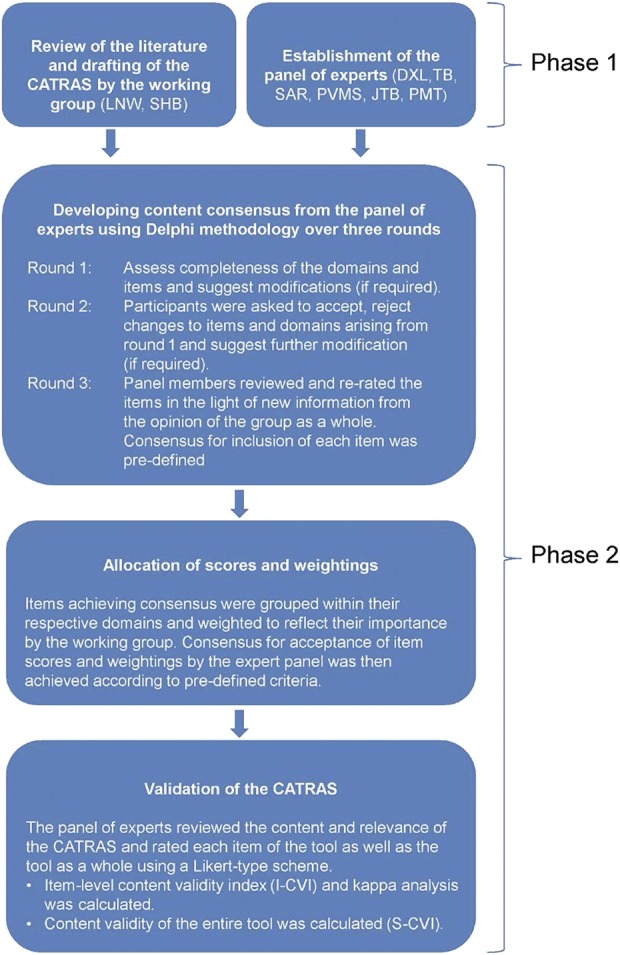
Diagrammatical representation of the sequence of tasks in this study. S-CVI, scale-level content validity index. LNW, Leon N. Warne; SHB, Sébastien H. Bauquier; DXL, B. Duncan X. Lascelles; TB, Thierry Beths; SAR, Sheilah A. Robertson; PVMS, Paulo V.M. Steagall; JTB, Juliana T. Brondani; PMT, Polly M. Taylor.

A working group identified and adapted potential domains and items to form the preliminary version of the CATRAS. The weight of each domain and associated item scores were assigned by the working group at this stage. This study reports the initial validation of the CATRAS by a panel of experts and comprised 2 phases: (1) development of consensus and preliminary agreement of content and (2) content validation. An overview of these phases is represented in Figure 1.

### 2.1. Identification and adaption of the domains and items

In 2014, the working group that comprised 2 of the authors (L.N.W. and S.H.B.) identified 3 domains, which would form the analytical scope of the CATRAS, and ultimately the framework used to critically appraise published analgesia studies.

(1) *Level of evidence* (CATRAS step 1, domain 1)—The working group adopted without modification, an LOE classification system that was previously used by a landmark systematic review initiative, the Reassessment Campaign on Veterinary Resuscitation (RECOVER). The LOE classification used by the RECOVER initiative was itself modified from a major human review group, the 2010 International Liaison Committee on Resuscitation (ILCOR 2010).^4,31^ This domain contained 6 items (LOE 1–6), which are characterised by criteria presented in Table 1, domain 1. The LOE of a study must be established before assessment of its methodological soundness.
(2) *Methodological soundness* (CATRAS step 2, domain 2)—The list of quality items contributing to this domain was adopted with minor modifications from that used in the RECOVER initiative process, which was originally derived from CATs designed by the OCEBM.^4,6^ Modifications included the addition of the following 2 quality item questions to each of the 5 possible categories (A–E): “*Was conflict of interest stated?*” and, “*Was the statistical methodology of the study appropriate? (If “NO,” please justify).*”
(3) *Grading of the PAT* (CATRAS step 3, domain 3)—The purpose of domain 3 is to provide critical appraisal of the PAT used in a study being evaluated. To assess the quality of the PAT used within an analgesia study being reviewed, domain 3 of the CATRAS requires the investigator to review the original or revised literature describing the development, refinement, or validation of the PAT. To achieve the third domain of the CATRAS, a CAT was developed based on a psychometric scoring system developed and validated to evaluate PATs used in noncommunicative critically ill human patients.^16^ The original psychometric scoring system used by Gélinas (2013) incorporated the GRADE system methodology.^16,21^ The content of the psychometric scoring system used by Gélinas (2013) was adopted largely unmodified from the original version, with only the following minor changes being made by the working group: substitution of the word “*scale*” for “*Pain Assessment Tool*” OR “*PAT*” to maintain continuity with nomenclature in other domains; all references to “*ICU patients*” were removed to maintain relevance to more than just ICU patients; anthropological examples used within the scoring legend of items within the original tool were substituted for species' neutral descriptors; a minor change was also made to the terminology of the question in item 3.5 from “*Discriminant validation*” to “*Sensitivity to change*” as the working group considered that this change provided greater clarity.

**Table 1:**
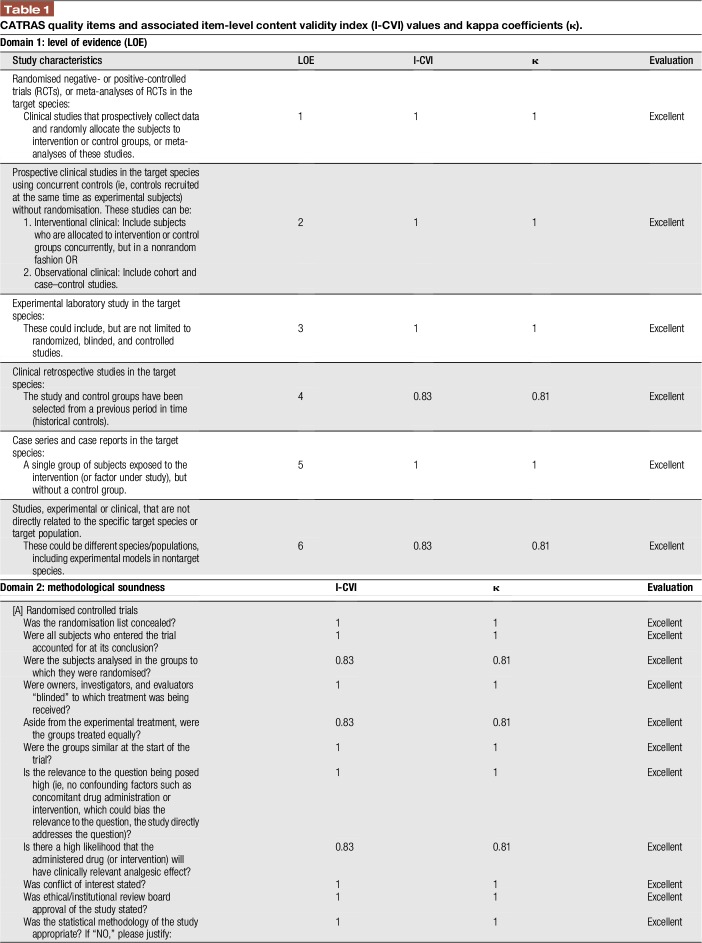

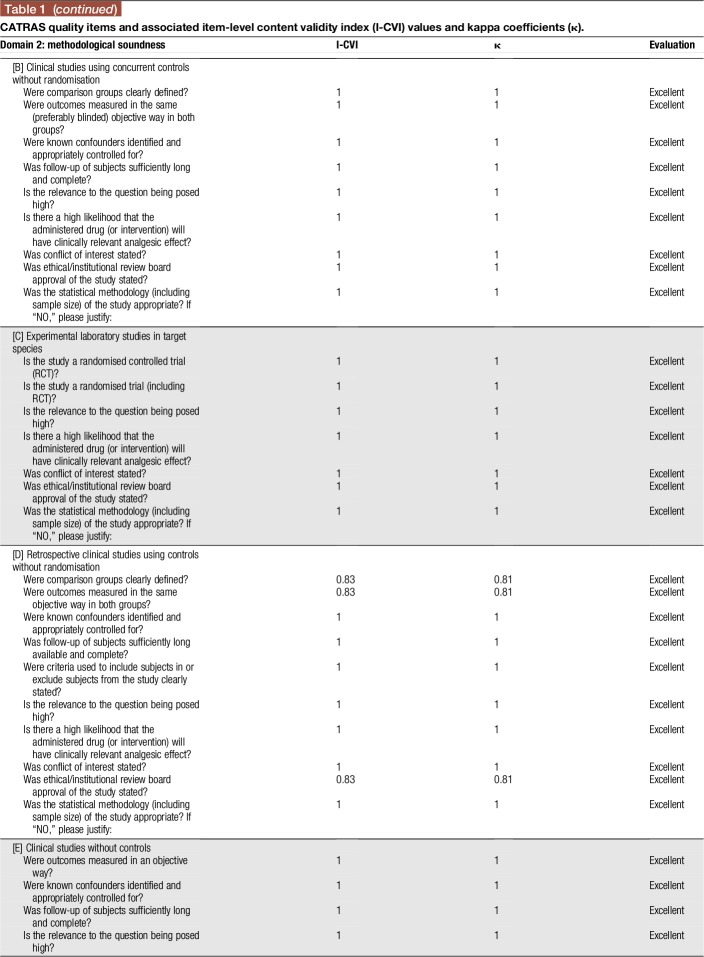

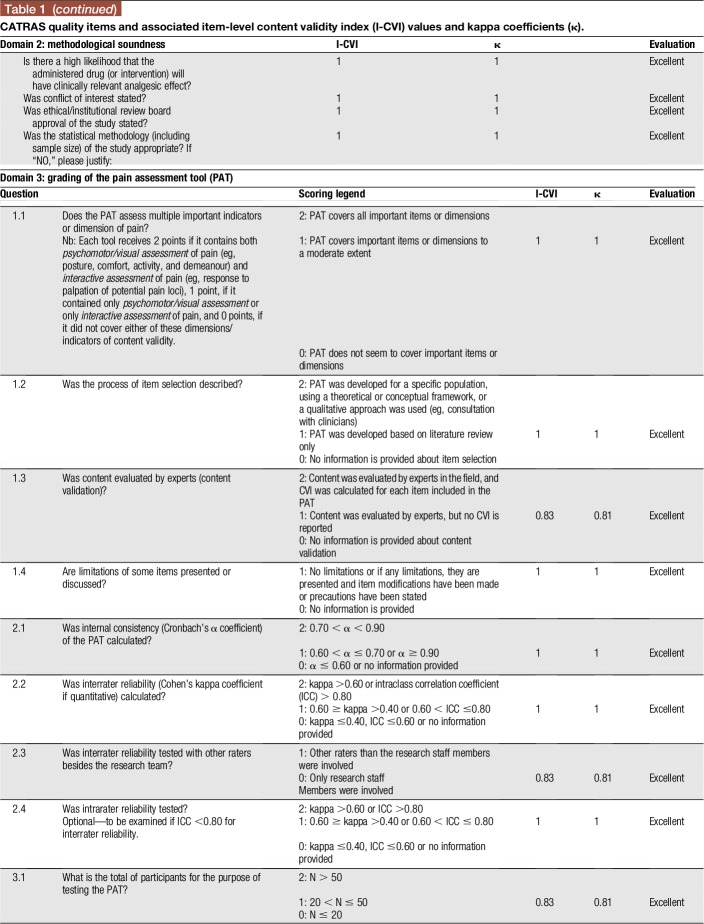

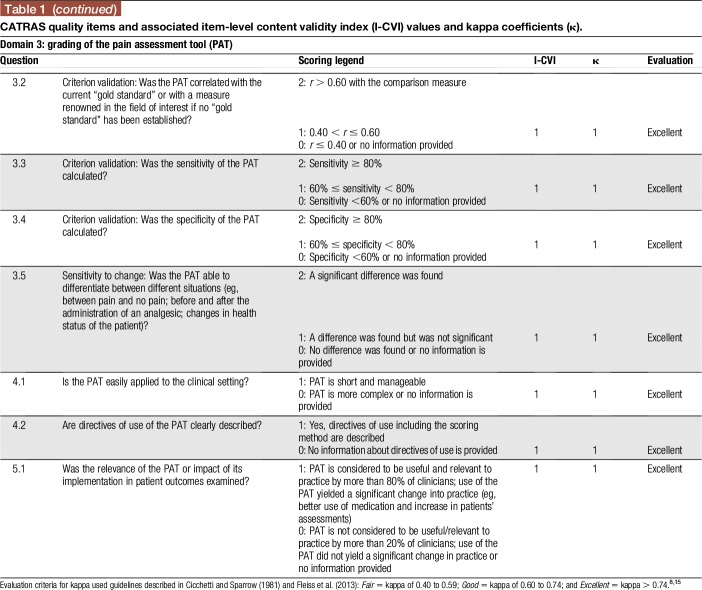
CATRAS quality items and associated item-level content validity index (I-CVI) values and kappa coefficients (κ).

### 2.2. Recruitment of the panel of experts

A panel of 6 internationally recognised experts from different institutions was established to critique the CATRAS. The expert panel comprised 6 of the authors (T.B., J.T.B., B.D.X.L., S.A.R., P.V.M.S., and P.M.T.). Panel members were selected based on their professional certifications and credentials, clinical experience and publication profile in translational research, veterinary pain management, and PAT construction.

### 2.3. Development of consensus

The study used Delphi methodology to develop consensus from a panel of experts, by means of surveys conducted over 3 rounds, to ensure that the 3 domains and items generated in the development of the CATRAS were not merely a function of the smaller working group by means of surveys conducted over 3 rounds.^22,29^ The objective of the first round was to gauge the completeness of the domains (and items within each) to assess adequately the quality of analgesia studies. Definitions for the domains and items within each were provided to enable comparison of each domain and item against its definition. Members of the expert panel were invited to contribute as many ideas as they wished in response to 2 open-ended questions regarding quality in analgesia studies: (1) “Are there additional domains beyond those already encompassed (ie, *LOE*, *methodological soundness*, and *grading of the PAT*), which you consider integral in comprehensively assessing the quality of analgesia studies? If YES, please list and explain your answer.” (2) Within the existing 3 domains, what factors not already encompassed by existing items (if any), do you consider important for assessing the quality of analgesia studies?”

In the second round, the responses obtained in round 1 were collated into one document by the working group and redistributed to the panel for individual rating of relevance as well as evaluation of the clarity of item construction and wording. Participants were asked to *accept*, *reject*, or *suggest modification to* points arising from round 1 relating to existing items or suggest additional items within each domain.

Information acquired from round 2 was then incorporated into the round 3 questionnaires with the addition of the participant's own ratings and comments for each item as a reminder. Thus, separate round 3 questionnaires were developed for each member of the expert panel. Panel members reviewed and rerated the items in the light of new information from the opinion of the group as a whole. Consensus for inclusion of each item was predefined as acceptance by 4 or more members of the expert panel (>4/6, >66%) without any further modification being recommended by any of the endorsing members. Any modifications were to be rated individually in a subsequent round, with consensus for inclusion being predefined as previously described.

### 2.4. Content validation

The panel of experts reviewed the content and relevance of the CATRAS and evaluated the appropriateness of each item of the tool as well as the tool's relevance as a whole using the following Likert-type scheme: 1 = not relevant, 2 = somewhat relevant, 3 = quite relevant, and 4 = very relevant.^28^

#### 2.4.1. Item-level content validity index and kappa analysis

For each item of the CATRAS, the content validity (item-level content validity index [I-CVI]) was calculated by dividing the number of experts assigning a rating of either 3 or 4 by the total number of experts—that is, the proportion of experts in agreement concerning the relevance. For example, an item rated as “quite relevant” or “very relevant” by 4 of 6 experts would have an I-CVI of 0.67.^37^ The kappa coefficient for individual items was also calculated using previously described methodology.^50^ Evaluation criteria for kappa used guidelines described in Cicchetti and Sparrow (1981) and Fleiss et al. (2013): *Fair* = kappa of 0.40 to 0.59; *Good* = kappa of 0.60 to 0.74; and *Excellent* = kappa >0.74.^8,15^ Items were considered to have adequate content validity for inclusion in the CATRAS if they achieved an I-CVI of 0.83 or greater and a kappa coefficient of 0.81 or greater. Kappa coefficients and I-CVI were calculated, and based on published recommendations, a cutoff point for an item to remain in the tool was predefined as 0.81 and 0.83, respectively (reflecting one disagreement).^30,37^

#### 2.4.2. Scale-level content validity index

The content validity of the tool as a whole (scale-level CVI [S-CVI]) was evaluated using previously described methodology, whereby the S-CVI is calculated as the average I-CVI across all items of the tool.^37^ Based on published recommendations, the minimum S-CVI required for the CATRAS to achieve content validity for the tool as a whole was predefined as 0.90.^37,49^

### 2.5. Development of the quantitative aspects of the assessment tool (CATRAS)

A final list of quality items that achieved consensus agreement from the panel of experts was collated by the working group. Items were grouped within their respective domains and weighted to reflect their importance within each domain.

(1) Domain 1—LOE: The highest score of 6 was attributed to the strongest LOE (LOE 1) and the lowest score of 1 to the weakest LOE (LOE 6) (Supplemental Table, available at http://links.lww.com/PR9/A29).
(2) Domain 2—Methodological soundness: The LOE of a study (step 1) must be established before assessment of its methodological soundness (Supplemental Table, available at http://links.lww.com/PR9/A29). Domain 2 consists of a list of methodological quality items (step 2 categories A–E) relating to the LOE assigned in step 1 (Supplemental Table, available at http://links.lww.com/PR9/A29). The overall methodological soundness is defined by 3 quality terms, as either: “good,” “fair,” or “poor.” Studies are to be assigned the quality-term “good” if they contain most or all the relevant quality items, “fair” if they contain some of the relevant quality items, and “poor” if they contain only a few of the relevant quality items but were considered to be of sufficient value to warrant inclusion in the next step of the review. The 3 quality terms “good,” “fair,” and “poor” were given a score of 3, 2, and 1, respectively.
(3) Domain 3—Grading of the PAT: Items were assigned weighted scores representing those ascribed by Gélinas.^16^

The sum total of all weighted items within each individual domain was transcribed into a percentage by dividing the attributed score by the maximum possible score of each respective domain. Transcription of the score into a percentage allowed for standardisation between the 3 domains of the CATRAS. Each domain was assigned equal weighting. Members of the expert panel were then asked to *accept*, *reject*, or *suggest modification* to the allocation of item scores and weightings. Consensus for inclusion of item scores and weightings were predefined as acceptance by 4 or more members of the expert panel without any further modification being recommended by any of the endorsing members. Any modifications were to be rated individually in a subsequent round, with consensus for inclusion being predefined as previously described.

## 3. Results

### 3.1. Evaluation for completeness and development of consensus

During the round 1 review of the draft CATRAS, no additional domains were deemed necessary by any member of the expert panel. The following item was added to the methodological soundness domain in each of the 5 possible categories (A–E): “*Was ethical/institutional review board approval of the study stated?*” This addition was unanimously accepted during rounds 2 and 3 of the review process, and no further items were modified or excluded.

### 3.2. Content validation

#### 3.2.1. Item-level content validity index and kappa analysis

The 67 items of the 3 domains were reviewed (Table 1). Fifty-seven (57/67; 85%) items received 100% agreement by all 6 members of the expert panel (I-CVI = 1; kappa coefficient = 1). Ten (10/67; 13%) items received 83% agreement (I-CVI = 0.83; kappa coefficient = 0.81).

#### 3.2.2. Scale-level content validity index

The content validity of the final remaining 67 items of the CATRAS resulted in a 97% (S-CVI = 0.97) agreement, indicating that the tool achieved *excellent* content validity.

### 3.3. Development of the quantitative aspects of the assessment tool (CATRAS)

After content validation, the working group assigned scores and weightings to the 67 quality items and the associated domains. The assigned scores and weightings for all 67 quality items and the associated domains were unanimously accepted without modification by the expert panel. The final results derived from application of the CATRAS are 3% scores (ie, one for each domain). The final version of the CATRAS is shown in the Supplemental Table (available at http://links.lww.com/PR9/A29). An example of the application of the CATRAS can be found in (Supplemental Appendix 1, http://links.lww.com/PR9/A22).

## 4. Discussion

In 2014, a working group (L.N.W. and S.H.B.) found no evidence of a published CAT designed specifically to evaluate the quality of published analgesia studies in any species. To address this absence, the authors designed and validated a 67-item CAT (Supplemental Table, available at http://links.lww.com/PR9/A29) that incorporates 3 domains (*LOE*, *methodological soundness*, and *grading of the PAT*).

Level of evidence is used by many review processes to create order and simplicity from the heterogeneity of published studies, and is assigned according to the study type and its inherent likelihood to exclude bias.^4,31^ The methodological quality and transparent reporting of an analgesia study is a key factor to consider when assessing its translational value.^36,41^ The quality items listed within the CATRAS to assess methodological soundness are primarily based on those used in the RECOVER initiative process, which were originally derived from CATs designed by the OCEBM.^4,6^ In addition, all the quality items described in methodological soundness category A of the CATRAS are part of the Consolidated Standards of Reporting Trials (CONSORT) 2010 “checklist of information to include when reporting a randomised trial.”^35^ The CONSORT statement was developed to improve the standard of reporting of randomised controlled trials for medical interventions.^3^ Furthermore, the methodological soundness domain of the CATRAS also complies with the Animal Research Reporting *In Vivo* Experiments (ARRIVE) guidelines methodology section, which highlights details of bias reduction tactics such as sample size calculation, random allocation to groups, and observer blinding.^25,41^ After recommendations from the panel of experts, the item “was ethical/institutional review board approval of the study stated?” was added to the methodological soundness domain (categories A, B, C, D, and E) both to strengthen the tool and to promote ethical research.

Content validity concerns the degree to which a scale has an appropriate sample of items to represent the construct of interest; that is, whether the domain(s) of content for the construct is adequately represented by the items.^37^ Content validity of the CATRAS was reviewed using a panel of 6 experts selected according to previously defined criteria.^12,18^ In addition, widespread geographical distribution of the panel members (Australia, Brazil, Canada, United Kingdom, and United States) allowed for differences in colloquial terms that could affect instrument comprehension by many diverse groups.^18^

There are potential biases in the methodology used. First, the statements are not an inventory of every aspect of methodology that could impact on trial quality. In an attempt to reduce the likelihood of this bias, the working group obtained consensus opinion from individuals with direct experience of conducting studies involving assessment of pain; as knowledge of the subject matter is considered the most significant assurance of a valid outcome using the Delphi methodology.^47^ Second, the reliability of the findings relating to validity coefficients may have been influenced by including the same individuals in both the content consensus and subsequently also as raters during the content validity process. The working group attempted to minimise this bias by both sequencing the order of the tasks and by their temporal separation: the consensus process occurred approximately 10 months before the content validity process.

A widely accepted method of quantifying content validity for multi-item tools such as CATRAS is the CVI based on expert rating of relevance.^37^ The CVI is an index of consensus and the extent to which experts share a common interpretation of the construct of a tool.^46^ A CVI was calculated for each quality item of the 3 domains (I-CVI) as well as for the overall CATRAS as the whole tool (S-CVI), thereby providing an index of interrater agreement. Critics of the CVI cite concerns about the possibility of inflated values because of the risk of chance agreement.^50^ In an attempt to address this issue, the current study used a previously described modified kappa-like index that adjusts each I-CVI for chance agreement or disagreement.^37^ Fifty-seven items received 100% agreement (I-CVI = 1); and ten items received 83% agreement (I-CVI = 0.83); there was no consistency observed in relation to individual members of the expert panel who rejected items of the CATRAS (including geographical location of the experts). Based on previously published guidelines citing the acceptable I-CVI in relation to the number of expert raters, these items of the CATRAS achieved adequate item-level content validity. In addition, using previously described evaluation criteria for kappa, these items were considered to have *excellent* agreement on relevance.^8,15^

Assessment of the S-CVI for the combined final 67 items of the CATRAS resulted in a 97% (0.97) agreement, indicating that the tool achieved *excellent* content validity. This result is considerably higher than published recommendations of the minimum S-CVI required for validation. Critical appraisal tool developers often set a criterion of 80% (0.80) or better agreement among expert reviewers as the lower limit of acceptability for an S-CVI.^11^ However, we chose to adopt the more stringent recommendations of Waltz et al.^49^ who set the lower limit of acceptable agreement at 90% (0.90).

It is widely accepted that interpretation of the results of a particular study should be informed by the quality of all aspects of the trial: the higher the quality, the greater the confidence in the validity and utility of the findings.^51^ When evaluating analgesia studies, the quality of the PAT used must be considered as a significant factor determining the quality of the report. Previously, consideration of the impact of the PAT on the strength of evidence has not been possible. Development of the CATRAS may now enable evaluation of the strength of findings from published analgesia studies based on a more thorough assessment of quality. The need to assess the original or revised literature for the purpose of grading the PAT may decrease the time efficiency of the CATRAS and might be considered a limitation by some users. Future work could streamline this process by establishing precalculated grades for the commonly used PATs. Another potential limitation of the CATRAS could be that because of the nonlinearity of both visual analogue scales and numeric rating scales, calculation of sensitivity and specificity (domain 3, item 3.3 and 3.4) may not be relevant for assessing these scales and as such they may be slightly downgraded by up to 4% of the overall possible score for domain 3. An interpretation of the results obtained from the grading of a PAT can be found in (Supplemental Appendix 2, http://links.lww.com/PR9/A23).

In evaluating the quality of published analgesia studies, it is important to consider both the analgesic efficacy and the safety of the drugs and regimens used.^9^ The CATRAS does not assess patient safety outcomes, and this may be considered a limitation.

In recent years, there has been growing interest in assessment of pain in animal subjects as well as in human patients incapable of self-reporting, with the number of newly developed PATs growing rapidly.^5,10,13,26,27,38,45^ It is the responsibility of researchers, funding agencies, and journals to prevent excessive growth of nonvalidated PATs in circumstances where appropriate and validated tools already exist. Further psychometric evaluation of existing PATs should be given priority over developing new tools for future use. Valid, practical, and reliable PATs can add to the body of knowledge about pain and help improve its treatment.

The final results derived from application of the CATRAS are 3% scores (ie, one for each domain). Illustration of these results should be at the discretion of the investigators; however, a radar chart would allow for a two-dimensional representation of the 3% scores on axes starting from the same point (Supplemental Appendix 1, http://links.lww.com/PR9/A22). Charting of the data in this way enables clear visual representation of the quality of a specific analgesia study in relation to the *LOE*, *methodological soundness*, and *grading of the PAT*.

Central to the practice of evidence-based medicine (EBM) is the process of asking well-focused questions, searching for the best available evidence, critically appraising that evidence for quality and validity, then applying the results to improve patient outcomes.^20,42^ The CATRAS is designed to facilitate the practice of EBM by enabling a quantitative quality assessment of an individual published study's evidence supporting (or rejecting) the clinical question being investigated. The CATRAS can be used to explore the influence of study quality or design methodology on the strength of the results and conclusions. We endorse the use of a systematic EBM approach that provides an explicit framework for formulating the clinical question or statement under investigation in terms of its 4 key parts—Problem/Population, Intervention, Comparison, and Outcome (PICO).^1^

The CAT developed in this study offers several benefits for assessing the quality of analgesia studies involving subjects incapable of self-reporting pain: its content was developed through the consensus of experts; it captures features of study design methodology, which are widespread in this field; and content validation has been established. The next step in the development of this important tool would be to apply the CATRAS in a systematic review of the literature focused on questions arising from analgesia studies.

## Disclosures

The authors have no conflict of interest to declare.

## Appendix A. Supplemental digital content

Supplemental digital content associated with this article can be found online at http://links.lww.com/PR9/A29, http://links.lww.com/PR9/A22, and http://links.lww.com/PR9/A23.
